# Emotional and Behavioral Problems in 4- and 5-Year Old Children With and Without Motor Delays

**DOI:** 10.3389/fped.2019.00474

**Published:** 2019-11-19

**Authors:** M. Christine Rodriguez, Terrance J. Wade, Scott Veldhuizen, Cheryl Missiuna, Brian Timmons, John Cairney

**Affiliations:** ^1^Faculty of Kinesiology and Physical Education, University of Toronto, Toronto, ON, Canada; ^2^Faculty of Applied Health Sciences, Brock University, St. Catharines, ON, Canada; ^3^Infant and Child Health Lab, McMaster University, Hamilton, ON, Canada; ^4^School of Rehabilitation Science, McMaster University, Hamilton, ON, Canada; ^5^Department of Pediatrics, McMaster University, Hamilton, ON, Canada

**Keywords:** developmental coordination disorder, CBCL, DSM, early childhood, preschool, comorbidity, co-occurrence, behavior

## Abstract

**Background:** An increased prevalence of psychological and behavioral conditions has been observed in youth and adolescents with DCD. The majority of research examining the relationship between motor skill proficiency and psychological problems has focused on older children and adolescents. The aim of the present study was to examine the relationship between motor skill proficiency and emotional and behavioral problems among pre-school age children with DCD to help determine how young children are when more severe problems begin to emerge (i.e., symptoms meet clinical thresholds) and the prevalence of comorbidity.

**Methods:** Children 4 to 5 years of age (*n* = 589) from the Coordination and Activity Tracking in CHildren (CATCH) study were divided into two groups: at risk for DCD (rDCD; *n* = 288) and typically developing (TD; *n* = 301). Inclusion in the rDCD group required a score ≤16th percentile on the Movement Assessment Battery for Children-2. Emotional and behavioral problems were assessed using the Child Behavior Checklist (CBCL) 1.5 to 5 year parent-report questionnaire. CBCL data were scored using the CBCL syndrome scales as well as the DSM V revised scale scoring.

**Results:** Seven children had missing or incomplete data on the CBCL and were excluded from the present analysis, leaving 582 participants. The mean age was 5.0 (SD 0.6) years and 57% of children were male (TD: 48% male, rDCD group: 67% male). After adjusting for sex, rDCD children scored significantly higher on all CBCL syndrome scales, all DSM-V scales, and all three summative scales. They were also significantly more likely to score at or above the syndrome scale clinical threshold on anxiety, withdrawn, emotionally reactive, aggression, ADHD, internalizing, externalizing, and total problems; and above the DSM-V thresholds on depression and autism. In addition, rDCD status was associated with a higher probability of meeting criteria for one, two, or more disorders in an ordinal logistic regression model.

**Conclusion:** Preschool-age children with rDCD have more parent-reported psychological problems, and are more likely to be above the clinical threshold for many psychological problems and meet criteria for multiple conditions.

## Introduction

Developmental coordination disorder (DCD) is a prevalent, neurodevelopmental disorder characterized by problems with fine and/or gross motor skills that are unrelated to other physical and/or intellectual impairments (1). DCD is thought to affect between 1.8 and 6% of children (1). Impairments in motor functioning are associated with attention, cognitive, social-emotional, and behavioral delays (1). Affected children also frequently present with poor physical fitness (2) and low physical activity (3), and are at increased risk of overweight and obesity (4).

Psychological and behavioral conditions associated with DCD in youth include both internalizing (e.g., depression/anxiety) (5–15) and externalizing problems (5–8, 12–14). Despite the recognized importance of the early childhood period for motor and psychosocial development, most research on these associations has focused on older children and adolescents. However, Livesey et al. (16) found that externalizing behaviors were related to ball skills in a small sample of 5- and 6-year-olds (*n* = 36), while MacDonald et al. (17) found that 3- to 5-year old children with lower object manipulation skill scores also demonstrated poorer social behaviors, including higher levels of externalizing/hyperactivity behavior. Given the difficulty of identifying emotional and behavioral problems in early childhood, some studies have instead elected to evaluate problem behaviors in the context of free play (18, 19). For example, Kennedy-Behr et al. (18) assessed aggressive incidents in preschool free play among 32 DCD and 31 matched children and found children with DCD significantly more likely to be both aggressors and victims.

While the majority of studies examining emotional and behavioral problems among children with motor coordination difficulties in early childhood have used observation or syndrome scales, an alternative is to use validated measures with clinical thresholds, such as the Child Behavior Checklist (CBCL). One study by Piek et al. (20) found that 3- to 5-year-old children at risk for DCD had significantly higher anxious/depressed subscale scores, but no difference in their withdrawn subscale scores, compared with their typically developing (TD peers). A larger study by King-Dowling et al. (21) examined 214 3- to 6-year-old children, 37 of whom were classified as having motor coordination difficulties. They found that children with motoric issues had higher levels of behavioral problems, with higher scores on the externalizing, aggression, and withdrawn subscales of the CBCL, but no significant difference in anxious/depressed nor overall internalizing behaviors. While there was a higher percentage of children with motoric deficits who scored above the clinical threshold in the total problem domain of the CBCL (8 vs. 3%), this difference was not statistically significant.

All studies to date that have examined preschool-aged children with motor coordination difficulties have used small samples (ranging from 7 to 37 children with DCD or at risk for DCD), a fact that may account for the variability in the significant associations reported. In addition, no previous work has examined clinical or subclinical thresholds or comorbidity. In addition, no study has examined the comorbidity of emotional and/or behavioral problems as a marker of severity of mental health problems among children at risk for motor delays (including DCD). The current study aims to extend the limited literature currently available and examine these relationships in the preschool period using a large sample of children at risk for motor delays. Specifically, this study will assess whether young children at-risk for motor delays have higher levels of emotional and behavioral problems, and whether their symptom levels are more likely to meet clinical thresholds for single and multiple emotional and behavioral problems.

## Materials and Methods

### Sample

The Coordination and Activity Tracking in CHildren (CATCH) study is a community-based, prospective cohort study of 589 children. Children were recruited from October 2013 to June 2017 in southern Ontario, Canada. The target sample size was 600 4- and 5-year-old children: 300 TD children and 300 children at risk for DCD (rDCD). Children and parents were eligible to participate if they could speak and read English and their children weighed more than 1,500 g at birth and did not have a diagnosed medical condition or physical disability affecting motor coordination (e.g., cerebral palsy or blindness). CATCH initially used two-stage screening, with parents first completing the Developmental Coordination Disorder Questionnaire (DCDQ) (22) over the telephone and then undergoing an initial selection before chosen individuals were invited to the laboratory to be assessed with the Movement Assessment Battery for Children, Second Edition (MABC-2) (23). Following the MABC-2 assessment, all children scoring at or below the 16th percentile and a random selection of children scoring above were invited to the longitudinal cohort. Due to the poor agreement of the DCDQ with the MABC, the process was modified (February 2015) to remove the initial screen. A higher-than-expected probability of motor impairment also necessitated changes to the randomization probabilities and processes to maintain approximate group balance. The screening and recruitment process is described more fully elsewhere (24). Ethical approval was obtained from the Hamilton Integrated Research Ethics Board at McMaster University. Informed, written consent was obtained from the parents of all participating children. This study examines the baseline data of the CATCH cohort.

### Measures

Motor coordination was assessed with the MABC-2, which comprises eight tasks assessing manual dexterity, ball skills, and static and dynamic balance. Raw scores on each task are converted into standard scores based on the child's age, and these scores are combined to produce an overall standard score and percentile. Children scoring at or below the 16th percentile (-1 SD) were considered to be rDCD and those scoring above the 16th percentile were considered TD.

Emotional and behavioral problems were assessed using the CBCL 1 ½ to 5-year parent-report questionnaire. The CBCL was developed to provide a standardized and detailed measure of dysfunctional behavior among children (25). The CBCL was completed by the child's parent or guardian; for 87% (509 of 582), it was completed by the birth mother. The CBCL consists of 99 statements describing a wide range of emotional and behavioral symptoms. Parents are asked to rate each statement on a 3-point scale (0–2), 0 being “not true” of the child, 1 being “somewhat or sometimes true,” and 2 being “very true or often true.”

CBCL subscales were developed to correspond to conditions in the 4th edition of the Diagnostic and Statistical Manual of Mental Disorders (DSM-IV) as well as empirically derived syndrome scales; however, a subsequent scoring revision by the developers, based on expert consultation, makes it possible to produce totals for disorder definitions from the DSM-V (26). Here, we report on the original set of syndrome subscales and the revised DSM-V scales. CBCL syndrome scale scoring produces eight subscales (anxiety/depression, withdrawn, emotional reactivity, somatic, aggression, attention, sleep, and “other problems”) as well as totals for internalizing (anxiety/depression, withdrawn, emotional reactivity, and somatic complaints) and externalizing (aggression and attention/ADHD) domains. DSM-V scale scoring includes five subscales: depression, anxiety, autism, ADHD, and oppositional defiant disorder (ODD). The CBCL syndrome scales have demonstrated strong criterion-related and construct validity (27). Reported reliabilities vary between 0.66 (anxiety/depressed) and 0.92 (aggressive behavior) for syndrome scales, between 0.89 (internalizing) and 0.95 (total) for the summative scales, and between 0.63 (anxiety) and 0.86 (ODD) for CBCL DSM-IV oriented scales (27). To date, two studies have provided preliminary validation for the CBCL DSM-V scales assessing autism (28) and ADHD (29).

### Statistical Analysis

We divided the sample into two groups based on measured level of motor proficiency: rDCD (MABC-2 percentile < = 16) and TD (percentiles 17-99.9). We identified children at risk for CBCL-measured conditions and calculated mean scores and percentages (prevalence) by group and sex. As the number of children scoring above the clinical thresholds for both the CBCL syndrome scales and DSM-V scales was relatively small, we used the subclinical threshold to identify children with possible problems. To test for differences by motor proficiency, we used multivariable linear, logistic, and ordered logistic regression, as appropriate, with group and sex as independent variables. We included sex because it is clearly associated with impaired motor functioning, and may also be associated with social-emotional problems. To identify possible *differential* associations by sex, we fit a further set of models including a group by sex interaction. To test independent associations between group membership and specific domains of functioning, we fit two logistic regression models with group membership as the dependent variable. In the first, we included gender and the internalizing and externalizing domain totals. In the second, we included gender and the five DSM-V subscales (which are fewer in number and capture broader areas of difficulty than the eight syndrome subscales). To obtain comparable coefficients, we standardized all subscales before fitting the models. We used Wald tests to obtain post-estimation comparisons of coefficients. We carried out all analyses for both syndrome scale and DSM-V categorizations of the CBCL subscales. Statistical significance was set at a *p* < 0.05, and all *p*-values were obtained from 2-tailed tests. We used Stata 14 (StataCorp) in all analyses.

## Results

The CATCH cohort contains 589 children (please see Cairney et al. (24) for further details of the full cohort). Seven children (1.2%) had missing or incomplete data on the CBCL and were excluded from the present analysis, leaving 582 participants. Of these, 320 (55.0%) were aged 4 and 262 (45.0%) were aged 5 years. Males comprised 57% (334 of 582) of the total sample. Based on their MABC-2 scores, 297 children (144 [48%] male, 153 [52%] female) were identified as TD and 285 (190 [67%] male, 95 [33%] female) as rDCD.

The results from the CBCL analysis using the syndrome scales are presented in Table 1 and compare rDCD and TD children both as a group and disaggregated by sex. After adjusting for sex, rDCD children scored significantly higher on all CBCL syndrome scales and all three summative scales (i.e., total problems, and internalizing and externalizing behavior). They were also significantly more likely to score at or above the subclinical threshold for anxiety/ depressed, withdrawn, emotionally reactive, aggression, and attention subscales, as well as the internalizing and externalizing behavior, and total problems. Finally, in an ordinal logistic regression model, rDCD status was associated with a higher probability for having a higher number of disorders. In a standard logistic regression, rDCD status was also associated with a higher probability of having multiple disorders, compared to having either a single disorder or no disorder at all (OR = 3.11, *p* = 0.002).

**Table 1:** CBCL syndrome scale scores and subclinical thresholds by group and sex.

	**Group**									
	**TD**			**rDCD**			**Total**			**Adjusted TD vs. rDCD difference (*p*)^**a**^**
	**Male (*n* = 144)**	**Female (*n* = 153)**	**Total (*n* = 297)**	**Male (*n* = 190)**	**Female (*n* = 95)**	**Total (*n* = 285)**	**Male (*n* = 334)**	**Female (*n* = 248)**	**Total (*n* = 582)**	
**CBCL syndrome scale scores (mean score [SD])**										
Anxiety	1.9 (1.9)	1.8 (1.6)	1.8 (1.8)	1.9 (2)	2.6 (2.2)	2.1 (2.1)	1.9 (2)	2.1 (1.9)	2 (1.9)	0.043
Withdrawn	1 (1.1)	0.9 (1.3)	1 (1.2)	1.8 (1.8)	1.8 (2)	1.8 (1.9)	1.5 (1.6)	1.3 (1.6)	1.4 (1.6)	<0.001
Reactive	2.1 (2)	2.1 (1.8)	2.1 (1.9)	2.6 (2.5)	2.7 (2.6)	2.7 (2.5)	2.4 (2.3)	2.3 (2.2)	2.4 (2.2)	0.002
Somatic	1.5 (1.5)	1.6 (1.7)	1.5 (1.6)	1.8 (2)	2 (2)	1.9 (2)	1.7 (1.8)	1.8 (1.8)	1.7 (1.8)	0.009
**Internalizing score**	6.5 (5.1)	6.4 (4.6)	6.5 (4.8)	8.2 (6.6)	9.2 (6.9)	8.5 (6.7)	7.4 (6)	7.5 (5.8)	7.5 (5.9)	<0.001
Aggression	7 (5.2)	6.1 (4.9)	6.5 (5.1)	9 (6.1)	8.1 (6.4)	8.7 (6.2)	8.1 (5.8)	6.9 (5.6)	7.6 (5.8)	<0.001
Attention	1.6 (1.5)	1.3 (1.5)	1.5 (1.5)	2.6 (2)	2 (1.9)	2.4 (2)	2.2 (1.9)	1.6 (1.7)	1.9 (1.8)	<0.001
**Externalizing score**	8.6 (6)	7.5 (5.8)	8 (5.9)	11.5 (7.3)	10.2 (7.6)	11.1 (7.4)	10.2 (6.9)	8.5 (6.6)	9.5 (6.8)	<0.001
Sleep	2.2 (2.2)	2 (1.8)	2.1 (2)	2.8 (2.5)	3 (2.7)	2.9 (2.6)	2.6 (2.4)	2.4 (2.2)	2.5 (2.3)	<0.001
Other	6.1 (4.2)	5.6 (4)	5.8 (4.1)	8.3 (5.7)	7.7 (5.8)	8.1 (5.7)	7.4 (5.2)	6.4 (4.8)	6.9 (5.1)	<0.001
**Total score**	23.4 (14.6)	21.4 (13.2)	22.4 (13.9)	30.8 (18.7)	30.1 (19.9)	30.6 (19.1)	27.6 (17.4)	24.7 (16.6)	26.4 (17.1)	<0.001
**CBCL syndrome scale subclinical threshold (%)**										
Anxiety	2.8	1.3	2.0	4.7	6.3	5.3	3.9	3.2	3.6	0.047
Withdrawn	0.0	1.3	0.7	7.9	5.3	7.0	4.5	2.8	3.8	0.002
Reactive	6.3	5.9	6.1	11.1	11.6	11.2	9.0	8.1	8.6	0.031
Somatic	6.3	7.2	6.7	9.5	11.6	10.2	8.1	8.9	8.4	0.116
**Internalizing**	2.1	1.3	1.7	7.4	8.4	7.7	5.1	4.0	4.6	0.002
Aggression	0.7	0.0	0.3	5.3	5.3	5.3	3.3	2.0	2.7	0.008
Attention	2.1	2.0	2.0	10.5	5.3	8.8	6.9	3.2	5.3	0.002
**Externalizing**	1.4	0.0	0.7	4.2	4.2	4.2	3.0	1.6	2.4	0.020
Sleep	4.9	0.7	2.7	4.2	8.4	5.6	4.5	3.6	4.1	0.094
**Total**	2.8	0.0	1.3	7.9	7.4	7.7	5.7	2.8	4.5	0.002
**Comorbidity of CBCL syndrome scale subclinical thresholds (%)**^**b**^										
0	88.2	85.6	86.9	71.6	73.7	72.3	78.7	81	79.7	<0.001^c^
1	7.6	11.1	9.4	17.4	15.8	16.8	13.2	12.9	13.1	
2+	4.2	3.3	3.7	11.1	10.5	10.9	8.1	6.0	7.2	<0.001^d^

*CBCL, Child Behavior Checklist; rDCD, At risk for developmental coordination disorder; TD, Typically developing; vs, versus*.

a *Adjusted for sex*.

b *Includes the CBCL subscale syndrome scores for internalizing and externalizing domains*.

c *Test of significance to assess the probability of having a higher number of disorders*.

d *Test of significance to assess the probability of having comorbid disorders compared to having either a single disorder or no disorder at all*.

Table 2 presents the data comparing rDCD and TD children on the DSM-V scales. Similar to the results observed with the syndrome scales, after adjusting for sex, children with rDCD scored significantly higher on all DSM-V scales. Children with rDCD were also significantly more likely to score at or above the DSM-V subclinical threshold for depression and autism. As observed with syndrome scales, children with rDCD also had a higher probability of having a higher number of disorders than TD children in an ordinal model, as well as a higher probability of having at least 2 or more comorbid disorders compared to having either a single disorder or no disorder at all (OR = 3.56, *p* = 0.007).

**Table 2:** CBCL DSM-V scale scores and subclinical thresholds by group and sex.

	**Group**									
	**TD**			**rDCD**			**Total**			
	**Male (*n* = 144)**	**Female (*n* = 153)**	**Total (*n* = 297)**	**Male (*n* = 190)**	**Female (*n* = 95)**	**Total (*n* = 285)**	**Male (*n* = 334)**	**Female (*n* = 248)**	**Total (*n* = 582)**	**Group difference (*p*)^**a**^**
**CBCL DSM-V scale score (mean score)**										
Depression	1.3 (1.5)	1.5 (1.5)	1.4 (1.5)	2 (2.1)	2.3 (2.4)	2.1 (2.2)	1.7 (1.9)	1.8 (1.9)	1.7 (1.9)	<0.001
Anxiety	2.5 (2.3)	2.2 (1.9)	2.4 (2.1)	2.6 (2.4)	3.3 (2.5)	2.9 (2.5)	2.6 (2.4)	2.7 (2.2)	2.6 (2.3)	0.006
Autism	2 (1.8)	1.6 (1.8)	1.8 (1.8)	2.9 (2.8)	2.6 (2.6)	2.8 (2.7)	2.5 (2.5)	1.9 (2.2)	2.3 (2.4)	<0.001
ADHD	3.1 (2.4)	2.8 (2.2)	2.9 (2.3)	4.3 (2.7)	3.5 (2.4)	4 (2.6)	3.8 (2.6)	3.1 (2.3)	3.5 (2.5)	<0.001
ODD	2.6 (2.3)	2.4 (2.2)	2.5 (2.3)	3.3 (2.7)	3.3 (2.5)	3.3 (2.7)	3 (2.6)	2.7 (2.4)	2.9 (2.5)	<0.001
**CBCL DSM-V subclinical threshold (%)**										
Depression	1.4	2.6	2.0	6.8	5.3	6.3	4.5	3.6	4.1	0.015
Anxiety	4.9	2.0	3.4	6.3	8.4	7.0	5.7	4.4	5.2	0.062
Autism	1.4	1.3	1.3	10.5	6.3	9.1	6.6	3.2	5.2	<0.001
ADHD	1.4	1.3	1.3	4.2	2.1	3.5	3.0	1.6	2.4	0.138
ODD	4.9	1.3	3.0	5.8	6.3	6.0	5.4	3.2	4.5	0.137
**Comorbidity of CBCL DSM-V subclinical thresholds (%)**										
0	90.3	90.4	90.4	78.8	81.1	79.5	83.7	86.9	85.1	<0.001^b^
1	5.5	7.1	6.3	10.9	13.7	11.8	8.6	9.6	9.0	
2+	4.1	2.6	3.3	10.4	5.3	8.7	7.7	3.6	5.9	0.020^c^

*CBCL, Child Behavior Checklist; DSM-V, Diagnostic and Statistical Manual of Mental Disorders, 5th Edition; rDCD, At risk for developmental coordination disorder; TD, Typically developing; vs, versus*.

a *Adjusted for sex*.

b *Test of difference in number of disorders*.

c *Post-estimation test for comorbid disorders versus either a single disorder or no disorder at all*.

Next, we fit a series of linear, logistic, and ordinal logistic regression models to compare the rDCD and TD groups across the CBCL syndrome and DSM-V scales and comorbidity while adjusting for sex (see Table 3). Overall, the rDCD group had significantly higher mean scores on all syndrome scales and DSM-V subdomains as well as on syndrome scale totals for internalizing, externalizing, and total scores.

**Table 3:** Linear and logistic regression analyses comparing rDCD and typically developing children to assess mean and subclinical threshold differences across CBCL syndrome scales and DSM-V scales adjusting for sex.

	**Mean scale differences^**a**^ (linear regression)**	**Subclinical Threshold^**a**^ (logistic regression)**
	rDCD Group	rDCD Group
	Coefficient (95% CI)	OR (95%CI)
**CBCL Syndrome Scales**		
Internalizing		
Anxiety	0.33 (0.01–0.65)^*^	2.68 (1.01–7.12)^*^
Withdrawn	0.79 (0.53–1.05)^**^	10.83 (2.49–47.2)^**^
Emotionally reactive	0.59 (0.22–0.96)^*^	1.96 (1.06–3.62)^*^
Somatic	0.40 (0.10–0.71)^*^	1.63 (0.89–2.98)
Internalizing score	2.12 (1.15–3.08)^**^	4.90 (1.81–13.27)^**^
Externalizing		
Aggression	2.01 (1.08–2.95)^**^	16.0 (2.08–123.01)^**^
ADHD	0.84 (0.55–1.14)^**^	4.25 (1.70–10.62)^**^
Externalizing score	2.86 (1.75–3.96)^**^	6.1 (1.34–27.84)^*^
Other domains		
Sleep	0.76 (0.38–1.14)^**^	2.12 (0.88–5.09)
Other	2.19 (1.37–3.01)^**^	–
Total score	7.92 (5.17–10.68)^**^	5.67 (1.91–16.82)^**^
Comorbidity^b^^,^^c^	–	3.11 (1.52–6.37)^**^
**CBCL DSM-V Scales**		
Depression	0.75 (0.43–1.06)^**^	3.26 (1.26–8.44)^*^
Anxiety	0.54 (0.16–0.92)^*^	2.12 (0.96–4.66)
Autism	0.97 (0.59–1.35)^*^	6.80 (2.32–19.92)^**^
ADHD	1.01 (0.60–1.41)^**^	2.46 (0.75–8.05)
ODD	0.81 (0.40–1.22)^**^	1.89 (0.82–4.36)
Comorbidity^b^	–	3.56 (1.41–9.00)^**^

*CBCL, Child Behavior Checklist; DSM-V, Diagnostic and Statistical Manual of Mental Disorders, 5th Edition; OR, Odds ratio; rDCD, At risk for developmental coordination disorder*.

* p < 0.05;

** *p < 0.01 (two-tailed)*.

a *Adjusted for sex*.

b *Compares the likelihood of scoring above the subclinical threshold on 2 or more subdomains*.

c *Includes the CBCL syndrome scores for internalizing and externalizing domains*.

For the subclinical threshold analyses using logistic regression, the rDCD group had a higher likelihood of meeting the threshold for all subdomains for the syndrome subscales with the exception of somatization and sleep. They were also more likely to meet the subclinical threshold for internalizing, externalizing, and total summative scores. For the DSM-V scales, the rDCD group were more likely to meet the subclinical threshold on depression and autism but not anxiety, ADHD, or ODD. Finally, for both the syndrome scales and DSM-V categorizations, the rDCD group were more likely to score above the subclinical threshold on two or more syndromes identifying a higher likelihood of comorbidity.

In the final analyses (not reported in tables), we examined group by sex interactions for the individual syndrome subscales and the comorbidity across subscales. The only significant group x sex interaction was for the CBCL anxiety syndrome scales, with the combination of female sex and rDCD status being associated with higher levels of anxiety on both syndrome scales and DSM-V subscales (syndrome scales, b = 0.79, 95% CI = 0.14 to 1.43, *p* = 0.017; DSM-V, b = 0.98, 95% CI = 0.21 to 1.75, *p* = 0.012). In addition, regressing group membership on internalizing and externalizing syndrome subscales showed that the association was somewhat stronger with external than with internal symptoms, but this difference was not significant (please see Supplementary Table 1). For DSM-V subscale totals, pairwise post-estimation comparisons suggest that group differences are significantly larger for autism (Chi Square = 4.68, *p* = 0.03) and attention (Chi Square = 3.92, *p* = 0.048) than for anxiety, though these contrasts should be interpreted with caution due to the number of comparisons performed.

## Discussion

The results of the study indicate that preschool age children with motor difficulties have more parent-reported emotional and behavioral symptoms than their TD peers. This was true of all CBCL syndrome scales as well as all three summative scales. These children were also more likely to be above the subclinical threshold on a wide range of psychological problems, and to meet CBCL subclinical thresholds for multiple conditions. These results provide evidence that young children with motor coordination difficulties may be experiencing more emotional and behavioral problems than previously recognized and a higher severity level than expected based on higher levels of comorbidity.

These results confirm findings from previous studies that have identified an association between motor impairment and externalizing behaviors in young children regardless of whether these studies used observational approaches or parent reports using validated instruments (16, 19–21). For example, two previous studies have shown that children with higher levels of motor skill are less likely to have externalizing problems. Livesey et al. (16) observed that higher ball skill scores were associated with lower externalizing behaviors on the Rowe Behavioral rating inventory in 5- and 6-year-old children. MacDonald et al. (17) found similar results in 3- to 5-year-olds: children with higher object manipulation skill scores demonstrated lower externalizing/hyperactivity behavior on the Social Skills Improvement System Rating Scale. Thus, it appears clear that there is an increased risk of externalizing behavior problems in preschool age children with motor coordination problems as it has been consistently observed in young children despite the different measures of emotional and behavioral problems used across studies.

Studies of older children and adolescents have consistently observed an association between poor motor skill proficiency and higher internalizing behaviors (5–7, 10–15), but only four studies have examined this relationship in young children (20, 21, 30, 31). Three of these studies observed higher internalizing problems in young children with poorer motor skill scores (20, 21, 30). Mancini et al. (30) found that lower motor skill scores were associated with greater internalizing problems on the SDQ. King-Dowling et al. (21) observed that children at risk for movement difficulties had significantly higher scores on the withdrawn subscale of the CBCL syndrome scale, but not on the overall internalizing subscale. In contrast, Piek et al. (20) found that children at risk for DCD had significantly higher scores on the anxious/depressed subscale but not on the withdrawn subscale (the internalizing total score was not evaluated). We found that rDCD children had significantly higher scores for all internalizing conditions, as well as on the internalizing subscale. Our larger sample size, and hence increased power to detect differences, is the likeliest explanation for differences between our study and previous research.

Unique to the present study, we also observed that rDCD children more often met subclinical thresholds for externalizing and internalizing behaviors on both the syndrome scales and DSM-V oriented scales; few TD children met these criteria. For example, for the aggression and withdrawn syndrome subscales, only 1 child and 2 children, respectively, scored above the threshold, compared with 15 and 20 children in the rDCD group. King-Dowling et al. (21), the only other study to utilize the CBCL to examine emotional-behavioral problems in young children, had very few cases meeting the clinical threshold on the CBCL and there were no significant findings. This contrasting result is likely attributable to the small number of children identified with motor difficulties (*n* = 37 vs. *n* = 285 in the present study).

Children with motor difficulties had more parent-reported emotional and behavioral problems than their TD peers on both the CBCL syndrome scales as well as the DSM-V oriented scales; however, while the observed pattern was similar, it was somewhat inconsistent between the two scoring methods. Children in the rDCD group scored higher on all CBCL syndrome scales, all DSM-V scales and all three syndrome scale summative scores; however, when examining scores meeting subclinical thresholds, rDCD children only scored at or above the clinical threshold for the DSM-V depression and autism scales compared with all syndrome scales (save somatic and sleep). The inconsistency is not surprising as the two scoring methods were created using different methods in order for the data to be used by both researchers and clinicians (32). The syndrome scale scores were empirically derived using large samples of children and using exploratory factor analysis and principal component analysis (32), while the DSM-IV, and later DSM-V, oriented scales were created through expert consensus (32, 33). The majority of studies done to date have compared the DSM oriented scales with syndrome scales for the CBCL 6-18, only de la Osa et al. (34) have conducted this comparison among preschool age children using the CBCL 1½-5. The authors compared the syndrome scale scores and DSM-V scale scores to the results of a diagnostic interview conducted with parents to examine their clinical utility (34). They demonstrated that, in general, the DSM-V scales did not perform better than the original syndrome scales when compared to the results of the diagnostic interview; however, specifically, only the DSM-V ADHD scale performed better than did the Attention problems syndrome scales. We observed a similar finding in the present study: rDCD children were significantly more likely to score at or above the syndrome scale clinical threshold for attention but not on the DSM-V ADHD scale. Despite these inconsistencies, together the results provide strong evidence that preschool age rDCD children have more emotional and behavioral symptoms than their TD peers on a wide range of psychological problems.

We examined the interactions between sex and motor functioning in this context. Previous studies have either not considered sex differences (16, 18, 19, 21) or included sex only as a main effect (17, 30, 31). We found a significant interaction for the anxiety subscale in both syndrome scales and DSM-V versions, with symptoms most common among rDCD girls. In our data, the overall rDCD-TD difference in anxiety is, in fact, attributable entirely to the difference among girls; these scores did not differ among boys. Given the large number of comparisons carried out, however, it is not clear whether a genuine sex-specific association between motor functioning and anxiety exists. No group by sex interaction was observed for externalizing behaviors. Overall, findings indicate that both boys and girls with rDCD have higher levels of internalizing and externalizing symptoms than their TD peers, while the possibility of a specific sex difference for anxiety deserves further consideration.

This is the largest study to date of the association between motor and social-behavioral problems among preschool age children. At the same time, there are limitations. First, these data are cross-sectional, so precedence is not clear. In addition, emotional-behavioral symptoms were only assessed by the parents (few others are well-placed to report on preschool-aged children). Despite these limitations, these results indicate that both the prevalence and severity of emotional and behavioral problems may be greater than previously thought for preschool age children, indicating that opportunities for interventions may exist at early ages. Results are also based on a community sample. Although this sample was a local community-based one, these results are more likely to generalize to the general population of pre-school age children with poor motor skills than would a research-based sample with children recruited in clinical settings.

In conclusion, this study addressed a knowledge gap in the literature by evaluating the severity of emotional and behavioral problems among preschool age children with poor motor skills. We observed that, compared with their TD peers, children with motor coordination difficulties, irrespective of sex, more often had emotional and behavioral problems, and more often had problems in multiple areas. Future studies should continue to be done to investigate whether these findings can be replicated and whether these differences are maintained as children age.

## Data Availability Statement

The datasets generated for this study are available on request to the corresponding author.

## Ethics Statement

The studies involving human participants were reviewed and approved by Hamilton Integrated Research Ethics Board, McMaster University. Written informed consent to participate in this study was provided by the participants' legal guardian/next of kin.

## Author Contributions

JC, CM, and BT were primarily responsible for the conception and design of the study. MR and TW drafted the manuscript. MR was primarily responsible for acquisition of the data. SV analyzed the data. All authors contributed equally to the interpretation of data, critical revision of the manuscript, read, and approved the final version of the manuscript.

### Conflict of Interest

The authors declare that the research was conducted in the absence of any commercial or financial relationships that could be construed as a potential conflict of interest.
