# Supplementary material for: Emotional and Behavioral Problems in 4- and 5-Year Old Children With and Without Motor Delays
Source: Front Pediatr. 2019 Nov 19;7:474. doi: 10.3389/fped.2019.00474 (PMC6877720; doi:10.3389/fped.2019.00474)
Supplement: Supplementary file 1 [file Table_1.docx]

**Supplementary Table 1. Logistic regression of rDCD group status on standardized CBCL internalizing and externalizing domain totals and DSM-V scales**

|  |  |
| --- | --- |
|  | rDCD Group |
|  | OR (95% CI) ^a^ |
| **CBCL Syndrome Scales** | |
| Internalizing | 1.21 (0.97 to 1.5) |
| Externalizing | 1.40 (1.13 to 1.74)** |
|  | |
| **CBCL DSM-V Scales** | |
| Depression | 1.26 (1.00 to 1.58)* |
| Anxiety | 0.94 (0.76 to 1.16) |
| Autism | 1.37 (1.10 to 1.71)** |
| ADHD | 1.29 (1.04 to 1.60)* |
| ODD | 1.02 (0.82 to 1.27) |

Note: CBCL=Child Behavior Checklist; DSM-V = Diagnostic and Statistical Manual of Mental Disorders, 5th Edition; OR = Odds ratio; rDCD = At risk for developmental coordination disorder

* *p* <0.05; ** *p* < 0.01 (two-tailed).

^a^ Adjusted for sex.
